# 3D multiscale analysis of the hierarchical porosity in *Coscinodiscus* sp. diatoms using a combination of tomographic techniques†

**DOI:** 10.1039/d1na00691f

**Published:** 2022-02-04

**Authors:** Othmane Darouich, Walid Baaziz, Dris Ihiawakrim, Charles Hirlimann, Danièle Spehner, Patrick Schultz, Hedwige Poncet, Virgile Rouchon, Sana Labidi, Corinne Petit, Pierre Levitz, Ovidiu Ersen

**Affiliations:** Institut de Physique et Chimie des Matériaux de Strasbourg (IPCMS), UMR 7504 CNRS, University of Strasbourg 23 rue du Lœss BP 43 67037 Strasbourg Cedex 2 France Ovidiu.Ersen@ipcms.unistra.fr; Institut de Génétique et de Biologie Moléculaire et Cellulaire (IGBMC), University of Strasbourg/CNRS UMR 7104, Inserm U1258 BP163 67404 Illkirch France; IFP Énergies Nouvelles, Rond-point de L'échangeur de Solaize BP 369360 Solaize France; Institut de Chimie et Procédés pour L'Énergie, L'Environnement et la Santé (ICPEES), ECPM, UMR 7515 CNRS, University of Strasbourg 25 rue Becquerel Cedex 02 67087 Strasbourg France; Laboratoire PHysico-chimie des Electrolytes et Nanosystèmes Interfaciaux, PHENIX, Sorbonne University, CNRS UMR 8234 75252 Paris France

## Abstract

A full 3D analysis of the hierarchical porosity in *Coscinodiscus* sp. diatom structures was carried out by using a multiscale approach that combines three advanced volumetric imaging techniques with resolutions and fields of view covering all the porous characteristics of such complex architectures: electron tomography, “slice and view” approach that uses a dual-beam microscope (FIB-SEM), and array tomography consisting of serial imaging of ultrathin specimen sections. This multiscale approach allowed the whole porosity network to be quantified and provided an unprecedented structural insight into these natural nanostructured materials with internal organization ranging from micrometer to nanometer. The analysed species is made of several nested layers with different pore sizes, shapes and connectivities and characterized by the presence of interconnected pores structured in various ways. The first evidence of the presence of a nanometric porosity made of ellipsoidal pores in the siliceous diatom frustules is also provided.

## Abstract 2

A multiscale 3D approach combining three advanced volumetric imaging techniques was employed for obtaining direct information on the hierarchical porosity in *Coscinodiscus* sp. diatoms having an internal organization ranging from micrometer to nanometer.

## Graphical abstract

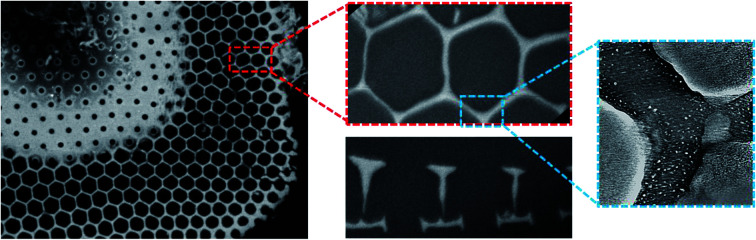

## Introduction

Porous materials have received increased scientific interest due to their peculiar properties^1–3^ and found applications in several fields such as materials engineering, bioengineering, petrochemicals, environmental protection, medicine and thermal insulation engineering^3–6^. Among the various types of porous materials which can be synthesized or directly encountered in nature, inorganic structures characterized by a hierarchical porosity display a growing interest due to their multiple advantages with respect to structures characterized by a unique porosity, in terms of the presence of a high porosity and specific surface area, a very good accessibility to the whole surface and a highly effective and selective diffusion towards the various molecular species used in applications.^7^ With pores distributed homogeneously or heterogeneously within the materials and with pores sizes of various dimensions, these materials are very appropriate for use in multiple applications in the fields of thermal insulation, energy, catalysis, filtering and storage^8–10^. Most of them do exhibit usually complex architecture with a wide range of pore sizes displaying both macropores and nanopores. These multi-scale structures can be used to protect or depollute groundwater, for the filtration of molecules, in petrochemistry, and in solute transport for example^11–15^.

Among the multi-scale and hierarchical porous structures found in nature, diatoms exhibit one of the highest long-range periodicities. These so-called living photonic crystals were observed for the first time under an optical microscope in 1702 by van Leeuwenhoeck.^16^ Their structure is made of an external siliceous skeleton with a complex and intriguing architecture called the “frustule”.^17^ From the point of view of the properties of interest, these self-assembled porous biosilica structures benefit from peculiar and very interesting properties in terms of photonic response, thermal stability and mechanical strength^18–21^. Several species of diatoms do exist with unit cells of various shapes and a wide range of pore diameters (from micrometer to nanometer) having typical sizes from that of large bacteria (size in the micrometric range), through intermediate ones which correspond to the size of a virus, to smaller sizes such as for instance those of nitrate molecules (in the nanometric range).^22–24^ They are nowadays deeply investigated due to their potential use as promising natural alternatives to synthetic porous silica for a wide range of applications including waste degradation, biomineralization-based processes or as bioindicators of water pollution in aquatic ecosystems.^25,26^ Recently, several theoretical and experimental studies were devoted to the investigation of the light trapping phenomenon in the diatom frustule in order to provide new solutions for the development of high-efficiency thin-film solar cells.^27^ Diatoms can also be used as catalytic supports, in particular for supporting palladium nanoparticles with potential use in the Heck and Suzuki reaction.^28^ In this general context, for paving the way of their subsequent use in applications, a comprehensive study of their porous architecture, in relation to their long-range order parameters, is mandatory. For example, the mass transport process that involves the biological or chemical systems mentioned before (viruses, bacteria, molecules…) into and out of diatom structures was found to be highly dependent on the internal structure and the porous architecture.^18^ A protocol is proposed by Mitchell to sort and treat different diatom species, according to their structural and morphological characteristics.^29^ In particular, one can mention a study carried out on two species of centric diatoms (*i.e. Coscinodiscus* sp. and *Thalassiosira eccentrica*) devoted to their ultrastructural description in which their potential applications as membrane filters is deeply discussed.^30^

Various techniques and methodologies are used to quantify the porosity and the porous network in nanomaterials. The most traditional ones consist in the measurement of the apparent porosity using gas or fluid absorption methods, which usually require drying the sample and can dramatically affect the materials structure during the analysis. Also, these techniques are based on the use of ideal geometrical models and assumptions, and are limited by their spatial resolution.^31^ The use of mercury intrusion porosimetry may provide open porosity characteristics in a pore range between 6 nm and 360 μm, as well as other types of information such as for instance the pore and particle entrance size distributions, pore shape and tortuosity.^32^ However, this technique is destructive, given that the mercury intrusion destroys the smaller pores as the pressure increases. In the same context, the BET (Brunauer–Emmett–Teller) technique enables the quantification of various parameters of the porous network such as the specific surface area, the pore size distribution and porous volume below 8 × 10^6^ nm^3^. However, the latter technique can be affected by diffusion problems through the small pores (<0.7 nm), which complicates the analysis of the measured data.^19,20^ An alternative solution is the use of other types of gases which are able to probe smaller pores. Among the techniques that are currently used for the analysis of the porosity of such structures, imaging methods are of particular interest since they provide a direct insight into their microstructure, some of them with very high resolution, in real space and without requiring specific models to be used as input in the data treatment. Many studies were based on the classical use of scanning electron microscopy (SEM) imaging for providing a direct insight into the diatom skeleton and its morphological features.^33,34^ More quantitative 3D information were obtained by using nano X-ray computed tomography (nano-XCT) allowing thus, for example, the internal structure of natural *Didymosphenia geminate* frustules to be determined.^35^ Regarding the diatom species of interest in this work, the frustule topographies of two types of diatom species (*Coscinodiscus* sp. and *Thalassiosira eccentrica*) were thus deeply investigated by atomic force microscopy (AFM)^30–36^. A 3D holotomography-based method was also used to obtain a full description of the 3D architecture of *Coscinodiscus* sp.^37^ Thus, one of the first applications of ion-abrasion scanning electron microscopy allowed the direct 3D perspective of the marine diatom *Thalassiosira pseudonana* to be obtained.^38^ Several studies were carried out using the FIB-SEM approach in the classical 2D imaging mode in order to obtain unprecedented information on the local porosity of a valve in *Coscinodiscus* sp. *frustules*^39^ or combined with the tomographic approach to yield long range and 3D information in diatomite specimens.^40^ However, all these methods and associated methodologies are able to provide structural and morphological information only for a specific range of pore sizes and the assessment of the hierarchical porosity which generally covers multiple ranges of scale is difficult. In this case, given the differences in the structural features of the analyzed fragments, even within the same specimen, the unique alternative allowing the hierarchical porosity at different levels and the connectivity between the pores to be quantified is the combined use of several techniques that are complementary with regard to their spatial resolution and fields of view and should be applied on similar fragments in a quantitative way.

In this general context, it is the purpose of the present work to propose such a combination of tomography techniques being able to perform the 3D analysis of the hierarchical porosity in diatom-based structures. They are: (i) electron tomography (ET), (ii) focused Ion Beam “slice-and-view” approach combined with Scanning Electron Microscopy (3D FIB-SEM) and (iii) array tomography consisting of sequential imaging by SEM of ultramicrotomy sections obtained in a serial way. Note that this last approach which used the ATUM-Tome set-up is applied for the first time, to the best of our knowledge, in materials science. Their combination provides a unique insight regarding the multiscale porosity and the 3D architecture in these natural specimens allowing the local features in terms of shape and connectivity of the pores, their spatial distribution as well as the periodicity and long-range characteristics of the porous network to be determined, all these parameters governing the potential use in applications of diatom species studied here.

## Results and discussion

### Long-range arrangement of the structural units using serial ATUM-SEM tomography

**Fig. 1:**
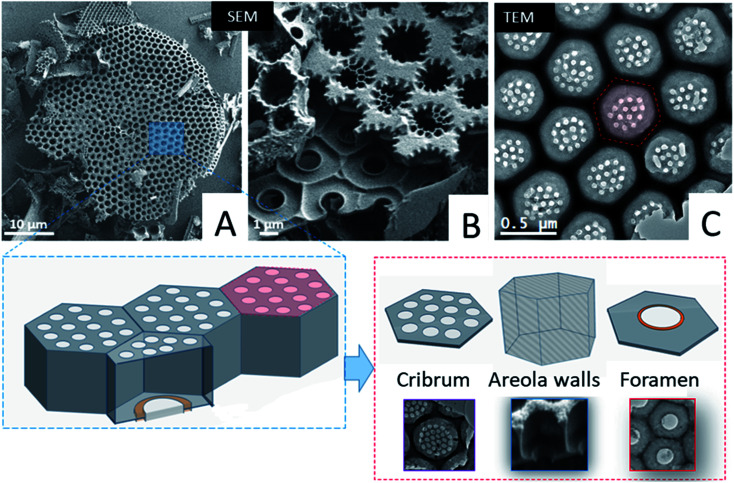
Top: representative SEM and TEM images acquired on typical fragments of the *Coscinodiscus* sp. diatom. (A) Global picture of a valve. (B) Picture of the two layers constituting the valve: the cribrum (top of the picture) and the foramen (bottom of the picture). (C) View of an intact part of the cribrum. On the lower part of the figure: sketches of the valve structure and the definition of the main parts: cribrum for the external layer of the valve, areola walls connecting the cribrum to the foramen, the internal layer of the valve.

First we studied the microstructure of *Coscinodiscus* sp. diatoms focusing on the long-range order within the porous structure in order to determine the spatial arrangement of the structural units and the periodical features of these 3D architectures. For that purpose, we transposed, for the first time ever, the ATUM-SEM tomography technique usually used in the field of biology. Diatoms are single-cell plants that do protect themselves from outside competitors by secreting a silica outer shell. The general shape of this shell, called the frustule, is that of two discoid valves with a diameter of approximately 40 μm, connected to each other by two lateral girdles. The whole shell has the general shape of a miniature pillbox. According to the literature the structure of the silica valves is threefold. The external layer, named the cribrum, is decorated with macroporous patterns of circular and/or elliptical shape with hexagonal symmetry between patterns. The internal layer of the valves, called the foramen, does have the same hexagonal symmetry and a unique large pore in its centre. The cribrum and foramen are connected by six intermediate walls with a honeycomb structure^38^ (see Fig. 1 and SI3†). A supplementary layer, called the cribellum, is sometimes reported in the literature^30^ but it is not visible in our studied fragments as it can be easily dissolved by the acidic treatment used to remove carbonate species or simply broken during the formation of the structures, due to its fragile character. This statement is supported by the work of Losic *et al.* that observed the degradation of cribellum layers which are only weakly bonded with the underlying silica layer.^30^

Typical large-scale SEM images (Fig. 1A, B and SI3†) do show a series of hexagonal patterned holes, which constitute the foramen layer and are maintained by the intermediate areolae layers with larger pore sizes which can be considered as the central part of the typical structural lattice. For the diatom species studied here, the foramen has the general appearance of a honeycomb network, with central holes having a roughly hexagonal shape. In addition, SEM and TEM images (Fig. 1) acquired on typical fragments do also illustrate the presence of some small circular pores (in the red hexagonal area drawn on the TEM image) which are localized on the other side of the main lattice of the structural lattice (called the valve), within the cribrum layer.

From a structural perspective, Gibaud *et al.*^37^ reported a 3D study by holotomography, which allowed the entire diatom frustule to be reconstructed and they also reported some morphological deformations of the areola walls. However, the study mainly provided global parameters such as for instance the mean diameters of the foramen holes. In this framework, our goal was to provide more detailed information regarding the spatial arrangement of the valves at short-range distance, between first order neighbors, and the spatial variation of some geometrical parameters that cannot be deduced from classical 2D observations. Such parameters are for instance the lateral size and the volume of the areola chamber both estimated from 2D images. They may vary at long distance, as it is also the case for the shape of the pores within the foramen layer which varies from place to place, even between neighbors, as it can be observed in the TEM image from Fig. 1 (note that the black area in this image can be assigned to the areola walls when they are visualized in projection).

**Fig. 2:**
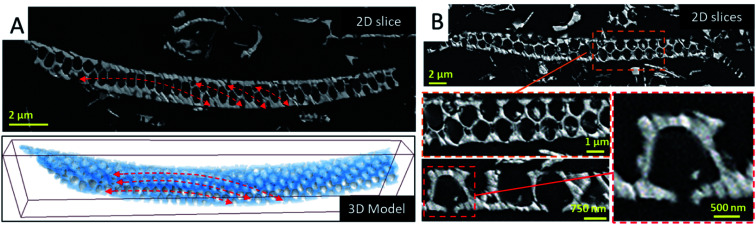
3D analysis of the diatomite structures by using array ATUM-SEM tomography. (A-top) Typical slice through a valve fragment illustrating the spatial arrangements of the structural units in curved layers underlined by red arrows. (A-bottom) 3D reconstruction of the same fragment. (B) Successive enlargements of a slice of a fragment of a *Coscinodiscus* sp. diatom valve down the areola chamber. The central hole in the foramen is clearly visible (bottom, right).

To analyze the long-range order of the structural units and the spatial variation of the geometrical parameters of the areola chambers, we analyzed several fragments by serial ATUM-SEM tomography (Fig. SI4).† The 3D analysis of a fragment of about 32 μm in length and 3 μm in thickness is illustrated in Fig. 2. By simply visualizing the reconstructed volume and the corresponding 3D model, two types of information are obtained. The first one is the spatial arrangement of the structural units which roughly correspond to the hexagonal prism represented in the bottom part of Fig. 1: they are circularly distributed on circle arcs, as shown in Fig. 2, in agreement with the macroscopic shape of the diatoms that is not delimited by planes but by curved surfaces. The second information we obtained by analyzing the slices extracted from the reconstructed volume is the shape of the areola chamber. Contrary to the cylindrical shape that was expected from 2D analyses it does exhibit a radial hexagonal symmetry in the plane parallel to the foramen and cribrum layers. Along the areola walls an anisotropic shape is observed: the thickness of the walls increases when going from the cribrum to the foramen.

From the 3D reconstructed volumes we could directly measure the mean “diameter” of the areola chambers taken at half distance between cribrum and foramen layers as well as the height of the chambers, the maximum distance between the cribrum and foramen. By measuring a series of a hundred chambers we could plot the histogram of their size distribution as shown in Fig. SI4.† A bimodal distribution can be assigned to the chamber size distribution with a minor peak close to 1.2 μm and a larger peak close to 1.5 μm. Our analysis also shows that, within our resolution limit of 100 nm given by the ultramicrotomy slice thickness, the height of the chambers is constant and equal to 1.2 μm. Therefore, the volume of the areola chambers varies slightly from one chamber to another and we could verify that this variation is quadratic with the mean “diameter” of the chamber. It is important here to underline that this heterogeneity in the geometrical parameters of the areola chambers is compensated by the shape of the areola walls and that it doesn't induce a significant disorder in their spatial, long-range arrangement. This can be observed in the large 2D view shown in Fig. 1. From a general point of view, such a finding highlights the ability of living organisms to compensate for local heterogeneities in the inorganic structures they construct and to reach a long-range order difficult to achieve by means of artificial synthesis methods.

### Local information obtained by FIB-SEM tomography

**Fig. 3:**
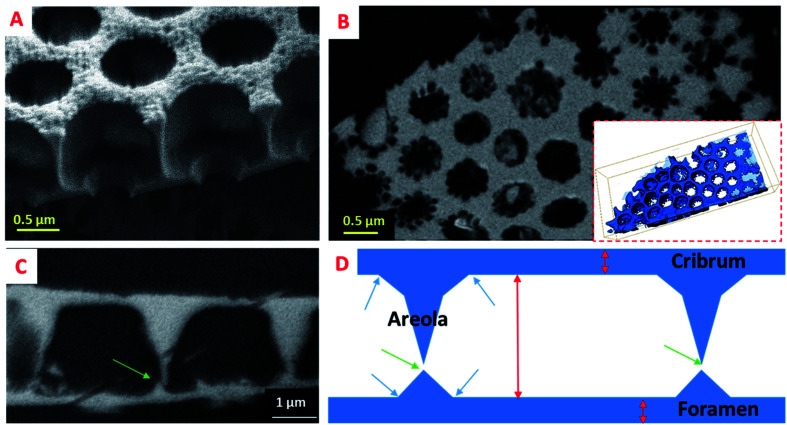
3D insight into the areola chamber of the diatomite structure. (A) SEM image of a broken fragment. (B) Longitudinal slice extracted from the FIB/SEM reconstructed volume, parallel to the cribrum and foramen layers, with the 3D model in insight. (C) Transversal slice, with the foramen layer in the bottom part. (D) Schematic representation of the structure illustrating the shape of the areola chamber, the positions we used to delimitate the three components (blue arrows), the height of the areola chamber and the thicknesses of the cribrum and foramen layers (red arrows), as well as the vertical positions of the connections between two succeeding areola chambers (green arrows).

**Fig. 4:**
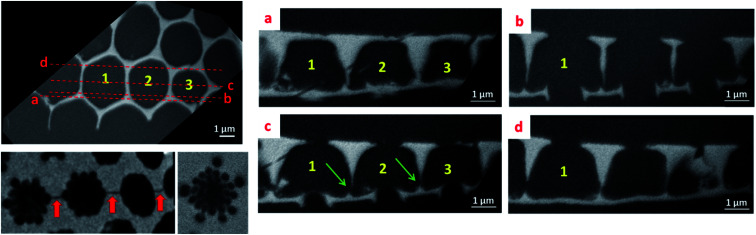
Left: longitudinal slices extracted from the reconstructed volume at the foramen (top) and cribrum (bottom) levels. The red arrows do indicate the connections between the central pores. Right: cross-sectional slices extracted from the reconstruction at the positions indicated on the longitudinal slice.

In order to obtain more resolved 3D information for assessing the morphology and the interrelation of the three valve components, the diatomite structure was studied using the “slice and view” FIB-SEM technique with a lateral and perpendicular sampling of 5 and 25 nm, respectively. From a quantitative point of view, such a 3D study allows us to determine and to compare the geometrical parameters of the three components. The protocol used to obtain the 3D model of the fragment under study from the 2D SEM images is illustrated in Fig. SI5† which shows some characteristic slices through several consecutive structural units. A simple visual analysis in the cross-section, perpendicular to the foramen and cribrum layers, provides several types of morphological information, with higher resolution as previously in the case of serial ATUM-SEM tomography. For instance, observation of cross-sections perpendicular to the valve layers shows a clear difference between the geometrical parameters of the foramen and cribrum layers, in terms of 2D structure, mean thickness and global porosity. First regarding the foramen, by considering the spatial delimitations between the components at the positions given by the inflexion points on the silica surface (arrows on the schematic representation in Fig. 3), we can determine its thickness which is about 180 nm. Analyzing the reconstructed volume slice by slice (longitudinal slices shown in Fig. SI5†) we can unambiguously solve its structure that consists of a periodical network of hexagons characterized by a quite irregular shape and a circular pore in their center with a mean size of about 1.2 μm. This value is in good agreement with that obtained from a 2D-based approach by Gilbault *et al.* (1.3 μm)^37^ and Losic *et al.* (1.15 μm).^30^ Another important structural detail which can be observed from the cross-sectional analysis (Fig. 4) is the presence of low dividing walls on the foramen layer close to the areola walls and on the borders of the circular pore. We measured their height to be about 230 nm, significantly larger than the values estimated by Losic *et al.*, by AFM, of about 75 nm.^41^ Except for these localized features, the 2D structure of the foramen does not change, as a function of the distance with respect to the areola chamber, and that leads to a global porosity of the foramen layer of about 33%, in agreement with the result found in the same study which reports a porosity of 35 ± 3%. Second, the thickness of the cribrum layer, measured using the same geometrical approach, is about 200 nm and its in-plane structure consists of a 2D arrangement of circular macropores with an average diameter of 300 nm. However, it can be reasonably assumed that, for higher lattice parameters of the structure, the number of the circular pores increases, without a direct influence on the global porosity. Regarding the porosity within the cribrum layer, we determined a value of about 27 ± 4% (see Fig. SI6†), slightly less than the one of the foramen. By applying the same method for the quantification of the porous volume to the areola chamber we found a value of 78 ± 5%; by combining the porosities of the three components, we obtained a total porosity of about 64% for the whole specimen.

One of the most important outcomes of this 3D analysis is related to the direct insight regarding the shape and the internal structure of the areola component which are difficult to solve otherwise, using only 2D observations. By analyzing the reconstructed volume slice by slice (Fig. 4), we can deduce the mean height of the areola chamber which is about 2.5 μm; however, the thickness of the walls delimitating the chamber, and consequently the width of the chamber, changes progressively with the distance to the foramen and cribrum layers. In particular, an obvious difference in the internal structure of the diatomite at the interfaces between the components can be unambiguously observed: the transition areas between the foramen and areola are made out of large pyramidal structures, which have triangular cross-sections, while at the interface between the areola and cribrum the contact areas are quite limited and no clear intermediate zone can be observed. A roughly trapezoidal shape can thus be assigned to the transversal section of the areola chamber, which has a radial symmetry along the vertical axis.

A last type of information which can be deduced from such a 3D analysis deals with the connectivity network between two neighboring structural units. In this regard, we can observe for the first time two types of small-sized connecting pores. The first ones are located within the cribrum layer and do connect two central pores, as observed in Fig. 4 (red arrows). The second ones do link the cavities of areola chambers, and they are located close to the foramen layers (green arrows in Fig. 3 and 4) and have a mean size of about 140 nm. Such a connecting network can be very useful for the subsequent use of these structures in some applications, as for instance the immobilization of large molecular structures needing to be accessible.

### Nanoporosity evidence by electron tomography

**Fig. 5:**
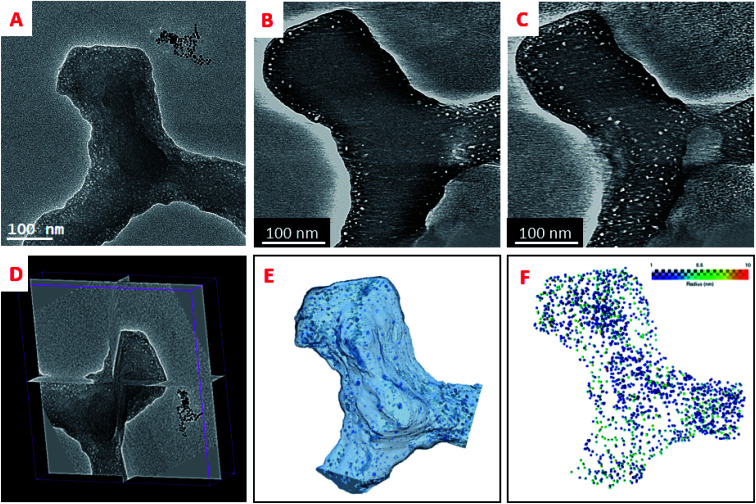
Electron tomography analysis of the diatomite structure. This specific object is at the crossing of three hexagons of the foramen. (A) 2D image of the analyzed fragment at 0° tilt, from a tilt series. (B) and (C) Typical slices extracted from the reconstructed volume parallel to the foramen and cribrum layers. (D) Three orthogonal slices crossing the center of mass of the analyzed fragment. (E) 3D model of the solid part obtained by data segmentation and of the sub-volume used for the porosity analysis; (F) 3D model of the pores illustrating their spatial localization within the grain.

To the best of our knowledge, the 3D analysis of the diatomite structure with a nanometer resolution has not been performed so far. In the following we performed a high resolution tomographic analysis on the specimen that was not embedded in a resin and thin fragments of the different parts, cribrum, areola and foramen were chosen for the 3D analysis. Fig. 5 illustrates the steps for such a tomographic analysis for a chosen fragment by using a tilt series, starting with the acquisition of the 2D projection images, the reconstruction step and the data segmentation that leads to the 3D model of the fragment. The general shape of the three constituents is in agreement with the one previously deduced by using other tomographic approaches (Fig. SI7†). The most important result we obtained is the evidence of the presence of a large number of pores in the solid part of the walls that can be easily observed by analyzing the reconstruction slice by slice. These pores have a mean diameter below 10 nm, they are present in the three layers and they seem to be distributed in the solid part preferentially close to the material surface. Once their contribution is extracted from the whole volumes by data segmentation, a more detailed analysis of their sizes, shapes and spatial localization can be performed, as presented below.

### Porosity distribution

**Fig. 6:**
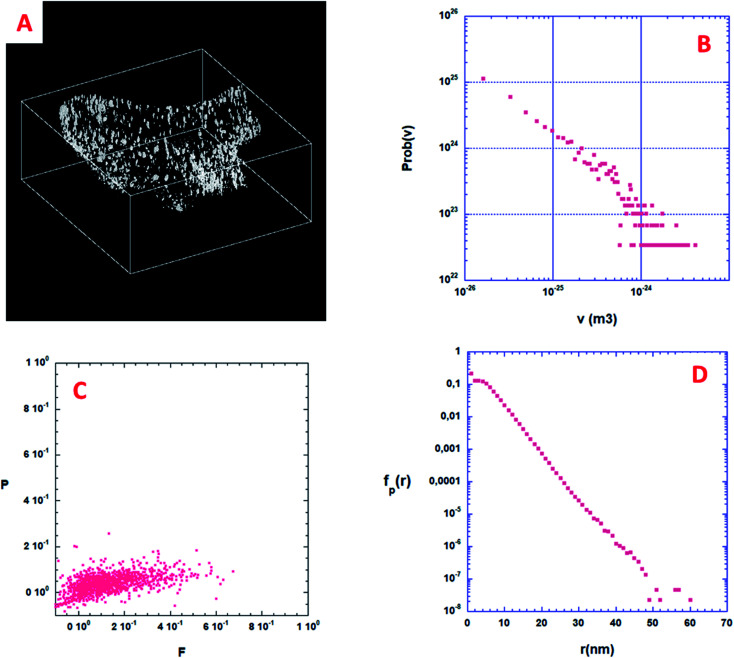
3D analysis of the nanoporosity within a volume extracted from the solid part of the diatomite. (A) 3D binary image of the nanopores; volume size = 804 × 770 × 330 voxels. A black voxel corresponds to void while a white voxel points out the presence of matter. (B) Probability density function Prob(*v*) of these nanopores. (C) Shape finder diagram associated with the isolated nanopores, see text for details. (D) Pore chord distribution function calculated using the binary 3D image shown in (A), see text for details.

From a quantitative point of view, the first information one can obtain is the volume distribution of individual pores, Prob(*v*), without making any assumption on their shapes. By considering all the pores shown in Fig. 6A, about 2000, Prob(*v*) is computed and shown in Fig. 6B. Most of the pores have a volume *v* between 10 and 1000 nm^3^. Assuming that a characteristic pore size is roughly equal to *v*^1/3^, the pore size spreads between 3 and 10 nm. A direct analysis of the 3D binary image shown in Fig. 6A shows that the volume fraction of the specimen corresponding to this nano-porosity is 1.922 10^−3^. Note that the resolution limit estimated here, given the experimental conditions used for the acquisition of the tilt series and specimen characteristics, is about 1 nm, meaning that all the pores with a size smaller than 1 nm cannot be determined.

### Shape distribution of the isolated pores

Some more information can still be extracted from the binarized data shown on Fig. 6A. Following Schmalzing *et al.*^42^ we will partly study the shape distribution of the isolated pores in the bulk by looking at their planarity and filamentarity. In three dimensions four Minkowski functionals, *V*_*μ*_, *μ* = 0, 1, 2, and 3 do provide a complete and unique description of a pattern of global morphology. We used these functionals to analyze the shape distribution and to decipher the morphological properties of these isolated nanopores looking for their flattening and elongation. According to Schmalzing: *V*_0_ = *V* (volume), *V*_1_ = *S* (external surface of the pore)/6, *V*_2_ = *H* (mean curvature)/3π, and *V*_3_ = *x*, *x* being the Euler characteristic of the problem. As we decided to only look at the flattening and elongation of the isolated pores then *x* = 1, we then constructed a shape-finder using the two factors *x* = π × *V*_0_*V*_2_/(4 × *V*_1_^2^) and *y* = 8 × *V*_1_*V*_3_/(3 × π × *V*_2_^2^), allowing the dimensionless planarity *P* = (1 − *x*)/(1 + *x*) and filamentarity *F* = (1 − *y*)/(1 + *y*) to be computed for each pore.

Fig. 6C shows a plot of the (*F*–*P*) diagram of all the pores in the sample that allows classifying the shape of these pores. In this diagram a flattened geometry is characterized by a small value of *F* meaning a vertical distribution. Inversely, an elongated shape distribution is associated with a small value of *P* and a horizontal distribution. As can be qualitatively observed in Fig. 6C, the experimental pore set has a global shape between a sphere and an elongated cylinder only slightly flattened. Such calculations strongly exemplify the characterization wealth that results from a quantitative analysis of the 3D microstructure of a porous sample.

### Specific surface of the isolated pores

When the considered pores of interest are not being connected, diffusion techniques are inefficient for estimating their specific surface in the material. To overcome this difficulty, we present here a technique based on the measurement of chord length distribution functions inside the 3D image of the material. A chord is a segment belonging to a pore and having both ends on the interface with the solid part. It can be considered as a linear path that correlates two distinct points of the interface. For a binary 3D image, like the one shown in Fig. 6A, there are two types of chords belonging either to the isolated pores (*p*) or to the complementary space that we will call the ≪solid≫ phase (*s*). From the 3D image it is easy to distinguish the two types of chords once the voxel separation between “void” and “matter” has been performed. According to Levitz and Tchoubar^43^ one can compute two probability density functions Prob_p_(*v*) for the pores and Prob_s_(*v*) for the solid phase, as a function of the volume of the pores. From these probability density functions one can calculate *f*_p_(*r*) and *f*_s_(*r*) measuring the size distribution of chords belonging to the pore or to the solid respectively as a function of their length. The chord length distribution *f*_p_(*r*) is shown in Fig. 6D. At large distance, it follows an exponential decay and its first moment 〈*l*_p_〉 is equal to 4.41 nm. For this very low porosity (1.922 10^−3^), the solid chord distribution *f*_s_(*r*) is difficult to compute and is sensitive to the finite size of the binary 3D reconstruction, and therefore less significant. Nevertheless, its first moment 〈*l*_s_〉 is well above 〈*l*_p_〉 as one would expect. One can estimate the specific surface of a nanopore as the ratio between its average surface *S*_part_ and its average volume *V*_part_. According again to Levitz and Tchoubar, the specific surface is directly related to the first moment through the relation *S*_part_/*V*_part_ = 4/〈*l*_p_〉. For the sample shown in Fig. 6A the analysis yields a value *S*_part_/*V*_part_ = 907 m^2^ cm^−3^, a unique quantity that cannot be reached using physical diffusion techniques.

### Angular distribution of isolated pores

In order to determine their structural order, we then focused on the measurement of the angular distribution of the isolated pores that are present in the sample reconstructed in Fig. 6A. As explained in a previous study,^44^ the 2D projection image *P*(*x*,*y*) of a 3D volume can be used to compute the 2D small angle scattering *I*(*q*_*x*_,*q*_*y*_) of the structure along the projection direction associated with *q*_z_. This is performed by using a 2D Fourier transform. This 2D scattering pattern can also be considered as a 2D spectral density, sensitive to the anisotropy of the structure. We have computed two projections, the first one, *P*_a_(*x*,*y*), along the thickness of the volume (having 330 pixels in depth), and the second one, *P*_b_(*x*,*y*), along the lateral direction (having 707 pixels in depth).

**Fig. 7:**
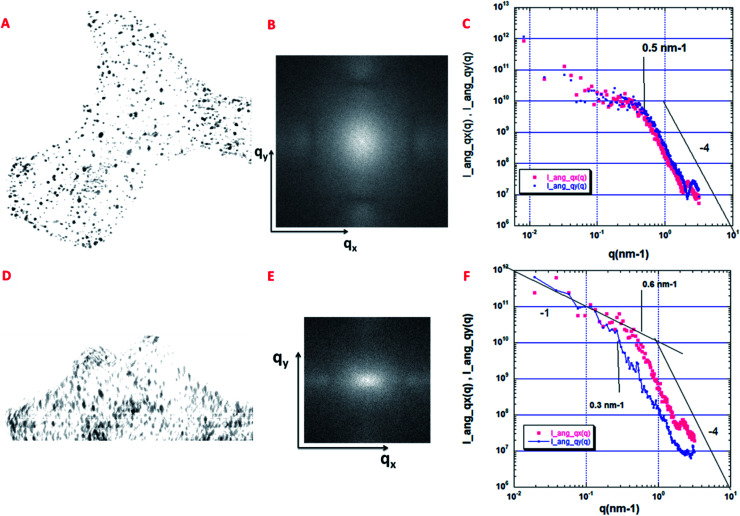
Quantitative analysis of the angular distribution of the pores. (A) Projections *P*_a_(*x*,*y*), along the thickness of the volume shown in Fig. 6A (330 pixels in depth); size 802 × 768 pixels, pixel size = 1 nm. (B) 2D small angle scattering *I*(*q*_*x*_,*q*_*y*_) associated with the *P*_a_(*x*,*y*) projection. (C) Small angle scattering curves along the *q*_*x*_ (*I*_ang_*q*_*x*_(*q*)) and the *q*_*y*_ (*I*_ang_*q*_*y*_(*q*)) directions after an angular azimuthal average of ±15^0^. (D) Projections *P*_b_(*x*,*y*) along the lateral direction of the volume shown in Fig. 6A (707 pixels in depth); size 802 × 768 pixels, pixel size = 1 nm. (E) 2D small angle scattering *I*(*q*_*x*_,*q*_*y*_) associated with the *P*_b_(*x*,*y*) projection. (F) Small angle scattering curves along the *q*_*x*_ (*I*_ang_*q*_*x*_(*q*)) and the *q*_*y*_ (*I*_ang_*q*_*y*_(*q*)) directions after an angular azimuthal average of ±15^0^.

As shown in Fig. 7A, the projection *P*_a_(*x*,*y*) along the thickness of the volume shows a distribution of isotropic spots with no global anisotropy. The same observation can be made in the reciprocal space (*q*_*x*_,*q*_*y*_) (Fig. 7B). More quantitatively, we have performed using Fig. 7B, two angular azimuthal averages of ±15^0^ along the *q*_*x*_ and the *q*_*y*_ directions respectively. This is shown in Fig. 7C where the two angular averages superimpose. Following,^44^ a nice q^−4^ Porod regime is observed above 0.5 nm^−1^, associated with a smooth pore surface (almost at the image resolution).

A different situation is encountered for the projection *P*_b_(*x*,*y*), along the lateral direction of the volume, as shown in Fig. 7D. First, we can clearly observe a global orientation of the elongated nanopores along the *y* axis. As shown in Fig. 7E, the 2D scattering *I*(*q*_*x*_,*q*_*y*_) is now anisotropic with a larger extension along *q*_*x*_. The two azimuthal averages are shown in Fig. 7F. They do not superimpose. The scattering curve along *q*_*y*_ is shifted to low *q*, meaning that we probe the largest dimension of elongated cylinders, which corresponds to the vertical axis, perpendicular to the three layers. The scattering curve along *q*_*x*_ is more sensitive to the smallest dimension of the elongated pores. Interesting enough, the end of the two Porod regimes, respectively 0.6 nm^−1^ for *q*_*x*_ et 0.3 nm^−1^ for *q*_*y*_ can be associated respectively with the characteristic distances *r*_min_ = π/0.6 = 5 nm and *r*_max_ = π/0.3 = 10 nm which could provide an estimation of the anisotropic pore sizes in good agreement with estimations given in the section dedicated to the analysis of the porosity distribution.

## Conclusion

By combining three different tomographic approaches, a multiscale 3D insight of the internal structure and hierarchical porosity of *Coscinodiscus* sp. diatom structures was obtained. The array tomography based on the sequential imaging of ultrathin sections obtained by ultramicrotomy was here used for the first time in the field of materials science and provided structural information on both the long- and short-range distances. It allowed us to observe that the structural units have the general shape of a hexagonal prism and that they are distributed on circle arcs; the areola chamber does exhibit a radial hexagonal symmetry in the plane parallel to the foramen and cribrum layers and has a variable width going from the cribrum to the foramen. At a lower scale, the “slice and view” FIB-SEM approach dealing with the use of a focused ion beam in scanning electron microscopy allowed us to obtain unprecedented information on the geometrical characteristics of all the present layers, foramen, areola and cribrum of the diatom, as well as to estimate their contributions to the global porosity of the specimen. A direct insight into the shape and the internal structure of the areola component was thus obtained, in particular the direct evidence of an intermediate region between the foramen and areola made out of large pyramidal structures as well as the presence of low dividing walls on the foramen layer close to the areola walls and on the borders of the circular pore. Finally, at the nanoscale, the 3D study by electron tomography evidenced the presence and allowed the reliable quantification of mesopores which are present in all the layers and have an elongated cylindrical shape with a preferential orientation of the long axis towards the macroscopic external surface of the specimen. From a general point of view, on the one hand, such a multiscale approach can be transposed for the study of any hierarchical system and, on the other hand, from the materials science perspective, our findings do pave the way towards the use of these diatomite structures in various applications.

## Materials and methods

### Diatomite specimens

The *Coscinosdiscus* sp. diatom species originate from a diatomite formation of the Sig Quarry, located in the Bas Chérif Basin in Algeria. The diatomite is formed mainly of centrophycidaes. These planktonic species lived in cold temperate waters during the Messinian period. In the raw sample, in addition to amorphous and organized silica frustules, quartz, calcite and oxide were identified by DRX. The crude diatomite is homogeneous in shade and light beige in color. The sample was then treated as follows: 5 g of crude diatomite was added to a 300 mL solution of 31% nitric acid by mass and the mixture is allowed to stand for about 17 hours. The sedimented diatomite solution was then centrifuged and washed to a neutral pH. 40 mL of distilled water is added to the solid deposit and the solution is allowed to decant for 13 minutes. The supernatant phase is then discarded. 40 ml of distilled water is again added to the decanted part and the mixture undergoes a second decantation for 1 minute. The liquid part contains the structures of interest; it is collected and dried out at 100 °C. The obtained dried powder is the primary source for the studied samples. The preparation protocol is summarized in Fig. SI8†.

### Specimen preparation for array tomography and 3D FIB-SEM

For 3D FIB-SEM analysis and ultramicrotome sectioning, the specimens under investigation needed to be mechanically strengthened and this was achieved by their impregnation with a resin. For the array tomography set-up using ultramicrotome sectioning, the specimen was embedded into epoxy resin according to standard protocols.^45,46^ For FIB-SEM analysis, the issues related to the sensitivity of the resin to electron and gallium atom irradiation should also be taken into account and the sample was embedded into an epoxy-Cure resin.^47^ The polymerization of the resins was performed under vacuum for 24 h at 2.5 bar at ambient temperature followed by a subsequent treatment at 70 °C for 12 h.

For the FIB-SEM analysis, the resin block was glued on an aluminum stub using silver paint (Silver dag 1415, Plano GmbH, Wetzlar). First all sidewalls besides the block face were covered with silver paint and then the block face was sputter-coated with a few nanometer thick platinum layer to avoid any charging of the resin. Prior to serial cross-sectioning and imaging no protection layer besides the sputter coating was deposited. An additional protective layer was deposited in the microscope using a distance between the gas nozzle and the grid center of about 2.5 mm. By opening the gas valve for 30 s a several 100 nm thick layer of platinum precursor was deposited. The preparation protocol is detailed in the ESI (Fig. SI9†).

### Protocol for obtaining the serial sections

To collect serial sections without critical artefacts, a preliminary trimming phase of the block face is required. We have used the trimming tool equipped with a binocular stereomicroscope to polish and form the upper block face and afterwards a razor blade to remove the part of the block which does not contain the diatom structures. Among the different block shapes tested (hexagonal, square, rectangular…), we have chosen the trapezoid shaped block that provides more regular sections, free of micrometric artefacts (Fig. SI1†). For the sectioning process, a diamond knife was used on a conventional ultramicrotome (PC PowerTome, RMC Product) with a speed of 2 mm s^−1^. After cutting, the successive sections floating on the surface of the water receptor have been collected simultaneously using the ATUM tool (RMC Boeckeler) on a ribbon tape. For each analyzed sample, we collected one hundred sections with a mean thickness of about 90 nm. The whole protocol, including also the data acquisition step, is summarized in Fig. SI2†.

### Acquisition of serial data for array tomography

The tapes containing the serial ultramicrotomy sections have been imaged on a scanning electron microscope. The individual SEM images have been acquired on a SUPRA40 microscope (Carl Zeiss Microsystems GmbH, Oberkochen, Germany) equipped with a field emission gun operated at a voltage of 5 kV and a HDAsB back scattered electron detector (BSE). The probe current was set to approximatively 5 nA and the magnification was chosen at 2.5 kX. The acquisition provided images of 1024 pixels × 768 pixels with a pixel size of 44.66 nm.

### Acquisition of the 3D data by FIB-SEM

FIB-SEM investigations were performed on an Auriga 60 Crossbeam (Carl Zeiss Microsystems GmbH, Oberkochen, Germany), a scanning electron microscope combined with a focused gallium ion beam column for serial FIB milling and imaging.

For 3D reconstruction 25 nm thick slices of the resin embedded sample were removed by FIB milling with a probe current set to 2 nA at 30 kV acceleration potential. The freshly exposed cross-section was imaged with a lateral pixel size of 5 nm in a serial manner. The FIB-milled cross-section width was 30 μm by 20 μm and the milling depth was 10 μm. The image store resolution was 2048 pixels × 1536 pixels resulting in an image width of about 10 μm and an image height of about 7.5 μm. For noise reduction purpose, line averaging with line averaging count number *N* = 11 and scan speed 4 was used. The SEM acceleration voltage was set to 1.5 kV, the SEM aperture was 60 μm and high current mode was turned on. For imaging the Energy selective Back-scattered electron (EsB) detector was used with a retarding EsB grid voltage of 1425 volts. The SEM imaging took about 29 s and the milling of each slice about 4 s. The acquired data cube consists of more than 400 single slice images. The grey level scale was inverted in order to obtain a TEM like image.

### Electron tomography

Electron tomography analyses were carried out on a JEOL 2100 FEG S/TEM microscope operated at 200 kV. 10 micrograms of diatoms powder were dispersed in ethanol and sonicated for 5 min. Then, five microliters of the prepared solution were deposited on the holey carbon membrane of a copper grid. A tilt series of 2D projected images were acquired using the tomography plug-in of Digital Micrograph that controls the specimen tilt step by step, the defocusing and the specimen drift. The individual TEM images, with a size of 2048 × 2048 pixels, were recorded using a CCD camera with a pixel lateral size of 0.15 nm. The specimen was tilted in the angular range of ± 76° using a fixed increment of 1.5°, giving thus a total of 100 images in each series. The recorded images were spatially aligned by cross correlating consecutive images using the IMOD software.^48^ The TomoJ plugin,^49^ integrated in the ImageJ software,^50^ was used to reconstruct the volume of the chosen part of the diatom structure by considering an algorithm based on the algebraic reconstruction technique (ART).^51^ Finally, the visualization and the analysis of the final volumes were carried out using the displaying capabilities and the isosurface rendering method in the Avizo software (FEI).^52^

### Data treatment: volume segmentation

The order of the image processing steps for the three tomographic approaches follows the same protocol. The tomographic reconstruction is firstly visualized and pre-processed using the free Fiji software library;^53^ depending on the type of reconstruction and on its characteristic signal to noise ratio, different filters are applied to reduce the noise and to improve the local contrast for being able to better detect and separate the outline of objects of interest. In a second step the image is segmented in order to extract quantitative information from the reconstructed volume. A semi-manual segmentation approach mainly based on classical threshold algorithms was preferentially used. The intensity threshold was chosen on the histogram of the whole volume which has a bi-modal distribution, with the pixel intensities clustered around two well-separated values, the first corresponding to the material and the second to the pores and the free space around the considered fragment. Once the structures of interest were extracted, their 3D visualization and modeling were performed for the quantitative assessment of morphological and topological parameters of interest in this study.

### Data treatment: quantification

In the binary volume obtained after segmentation, the porous phase is represented by black voxels and the material one by white voxels. For reducing the contribution of the noise, we applied several erosion/dilation dual operations to the binary volumes.^54^ For measuring individual parameters such as the mean pore size of the chambers or their corresponding volumes, the pores should be geometrically separated, even if they are connected through smaller pores or throats; for this purpose, the watershed algorithm was systematically used which diminishes the influence of the contact points. The pores were afterward labeled and ordered in a series which was subsequently used in the morphological analysis. Various parameters have been quantitatively determined for providing a full insight on the hierarchical porosity. The first class concerns the local parameters, in particular the general shape of the pore, their 3D mean size and their interconnections; these parameters can also be determined statistically by averaging the values obtained on all the pores present in the analyzed volume. The second set of porous characteristics concerns the specific surface area and the porous volume which can be considered as classical parameters, traditionally used for the characterization of any porous specimen. The porous volume is defined by the ratio between the number of voxels located within the pores and the total number of the voxels. The specific surface area is defined as the surface area induced by the presence of pores per unit of solid volume of porous material and can be measured as the ratio between the surface and the mass of the considered grains. Other more complex parameters that can be useful for the description of the thermal properties, are the pore size distribution (PSD), the shape of the pores and the chord length distribution.

## Author contributions

O. D., D. I., W. B. and H. P. carried out the experiments. C. P. and S. L. prepared the diatom specimens. P. S., D. S. and V. R. participated in the acquisition of the FIB-SEM and SEM data. O. D., P. L., C. H. and O. E. interpreted the data and wrote the manuscript. O. E. and D. I. conceived the original idea. O. E. and W. B. supervised the project. All the authors participated in the interpretation of the results, critical feedback, analysis and editing of the manuscript.

## Conflicts of interest

There are no conflicts to declare.

## Supplementary Material

NA-004-D1NA00691F-s001
