# Supplementary material for: 3D multiscale analysis of the hierarchical porosity in Coscinodiscus sp. diatoms using a combination of tomographic techniques
Source: Nanoscale Adv. 2022 Feb 4;4(6):1587–98. doi: 10.1039/d1na00691f (PMC9418519; doi:10.1039/d1na00691f)
Supplement: NA-004-D1NA00691F-s001 [file NA-004-D1NA00691F-s001.pdf]

Electronic Supplementary Material (ESI) for Nanoscale Advances.
This journal is © The Royal Society of Chemistry 2022

SUPPORTING INFORMATION

3D multiscale analysis of the hierarchical porosity in Coscinodiscus sp. diatoms using a combination of tomographic techniques

Othmane Darouich[1], Walid Baaziz[1], Dris Ihiawakrim[1], Charles Hirlimann[1], Danièle Spehner[2], Patrick Schultz[2], Hedwige Poncet[3], Virgile Rouchon[3], Sana Labidi[4], Corinne Petit[4], Pierre Levitz[5], Ovidiu Ersen[1*]

1- Institut de physique et chimie des matériaux de Strasbourg (IPCMS), UMR 7504 CNRS, University of Strasbourg, 23 rue du Lœss BP 43 , 67037 Strasbourg Cedex 2, France.

2- Institut de génétique et de biologie moléculaire et cellulaire (IGBMC), University of Strasbourg/CNRS UMR 7104, Inserm U1258, BP163, 67404 Illkirch, France.

3- IFP Énergies Nouvelles, Rond-point de l'échangeur, BP 3,69360 Solaize, France.

4- Institut de Chimie et Procédés pour l'Énergie, l'Environnement et la Santé (ICPEES), ECPM, UMR 7515 CNRS, University of Strasbourg, 25 rue Becquerel Cedex 02, 67087 Strasbourg, France.

5- Laboratoire PHysico-chimie des Electrolytes et Nanosystèmes InterfaciauX, PHENIX, Sorbonne University, CNRS UMR 8234, 75252 Paris, France.

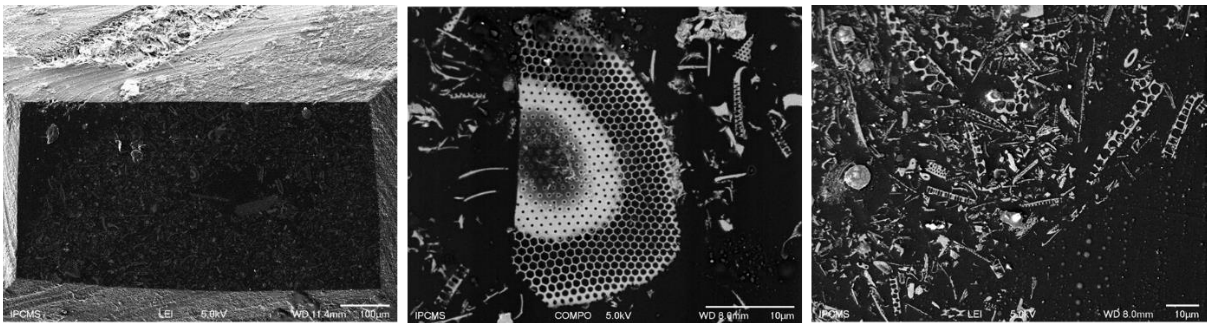

Figure SI1: SEM images of the block obtained by embedding the diatoms structures into an epoxy resin.

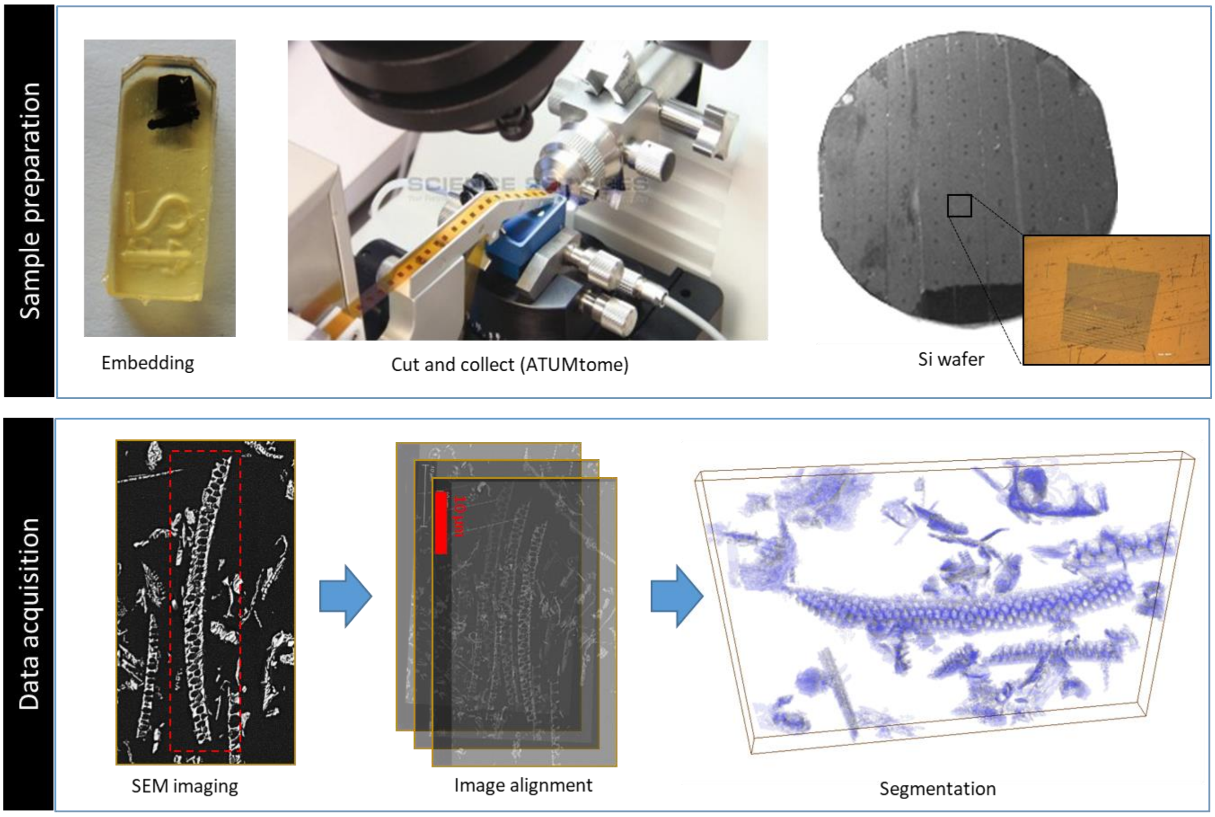

Figure SI2. Illustration of the global approach used in serial ATUM-Tome tomography.

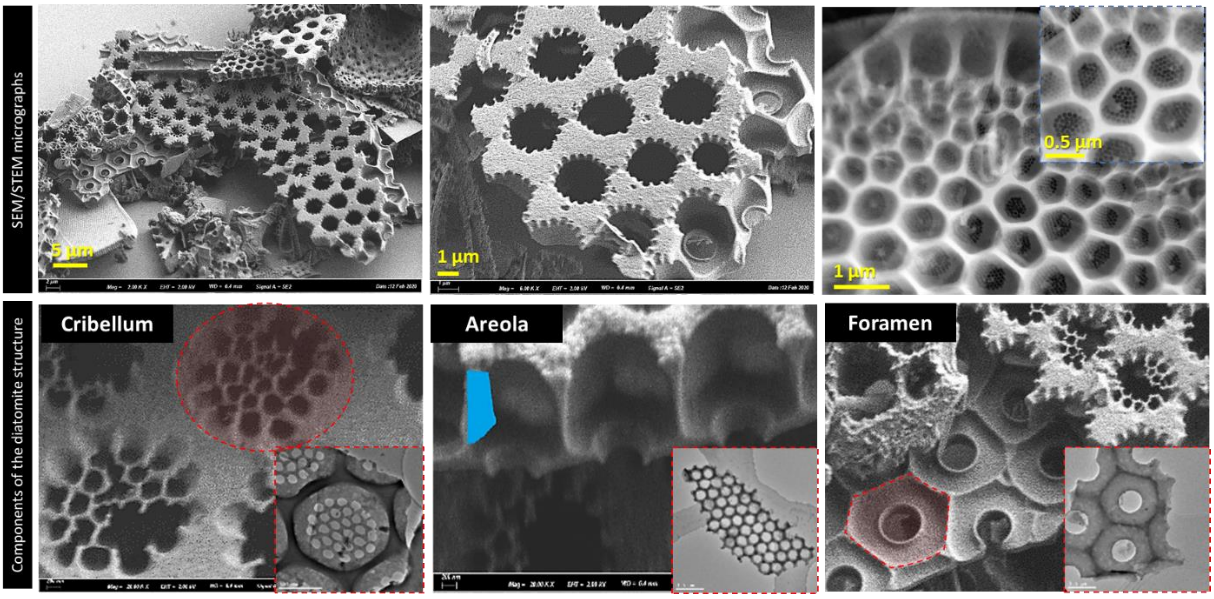

Figure SI3. Typical SEM and STEM images of the considered diatomite structure, acquired in various parts of the structures. The red circled areas represent the structural units of the cribellum and foramens layers and the blue colored area (middle-bottom image) illustrates the wall of an areola chamber.

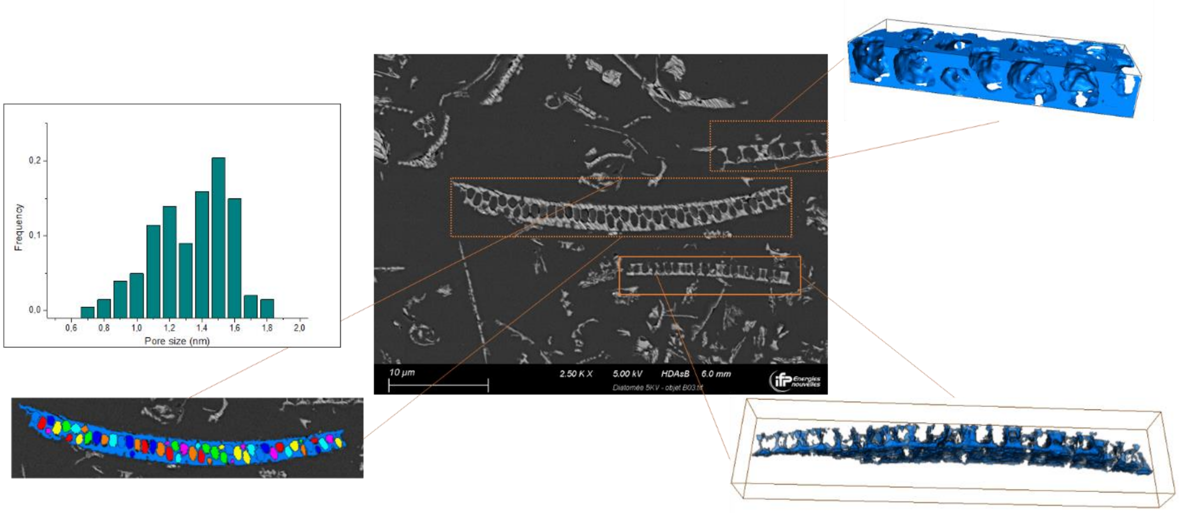

Figure SI4: Illustration of the modelling and quantification protocols used in serial ATUM-Tome tomography.

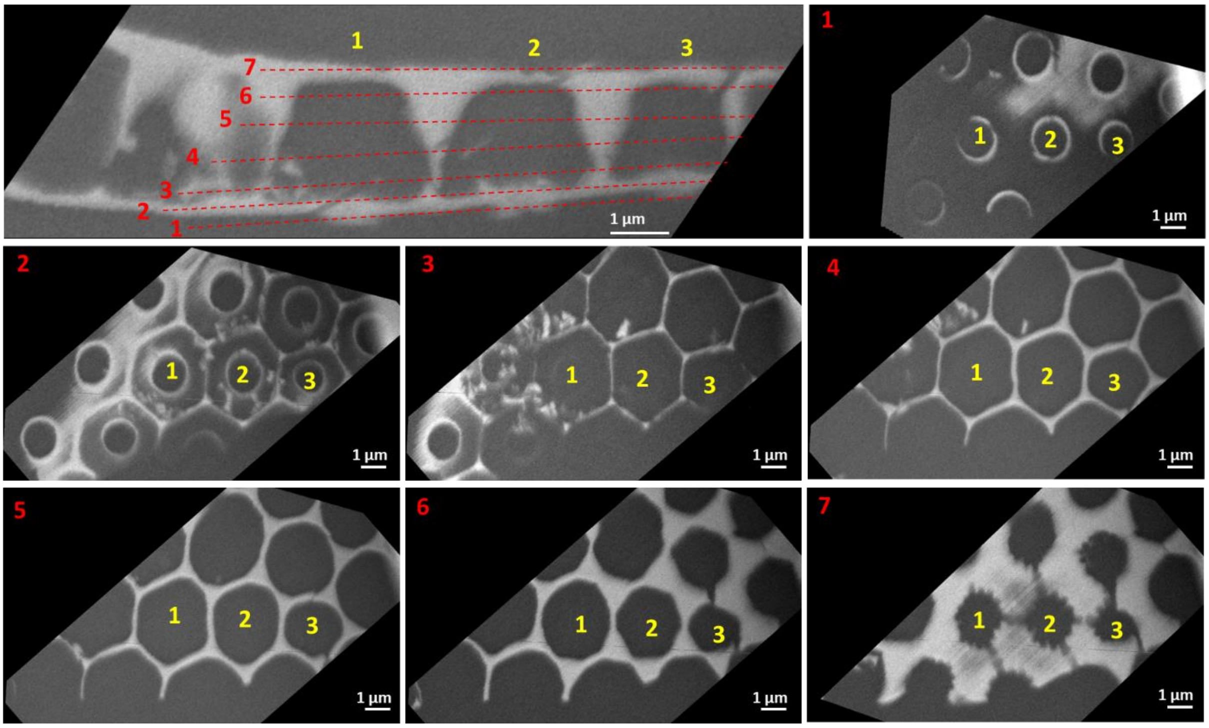

Figure SI5: Typical longitudinal slices obtained by FIB-SEM tomography for the diatomite structures.

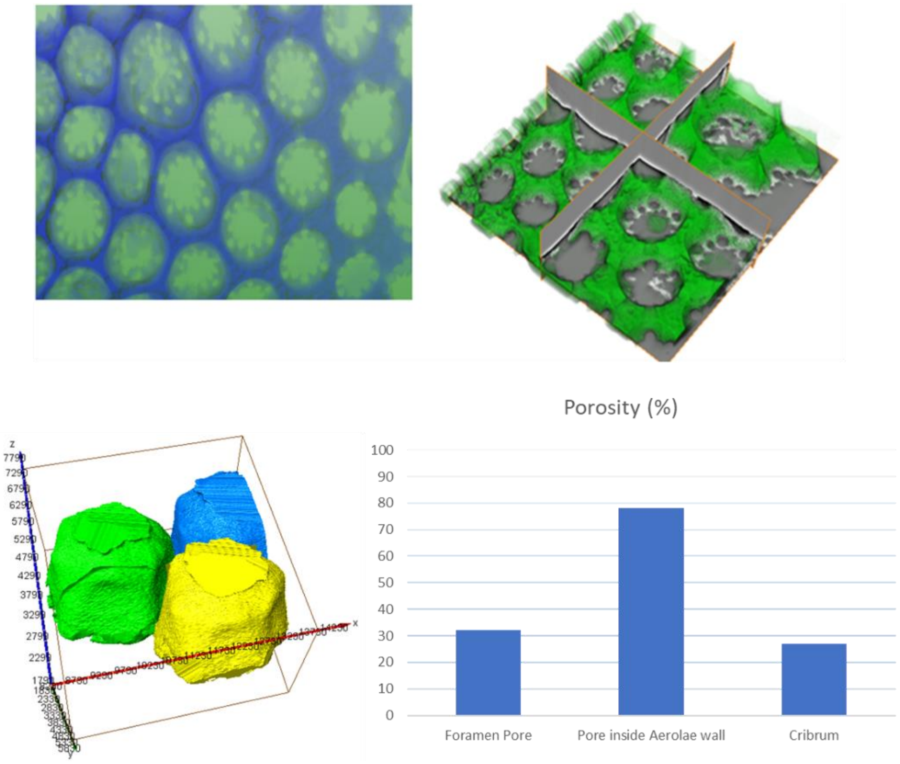

Figure SI6: Illustration of the protocol used for the quantification of the volumes obtained by FIB-SEM tomography

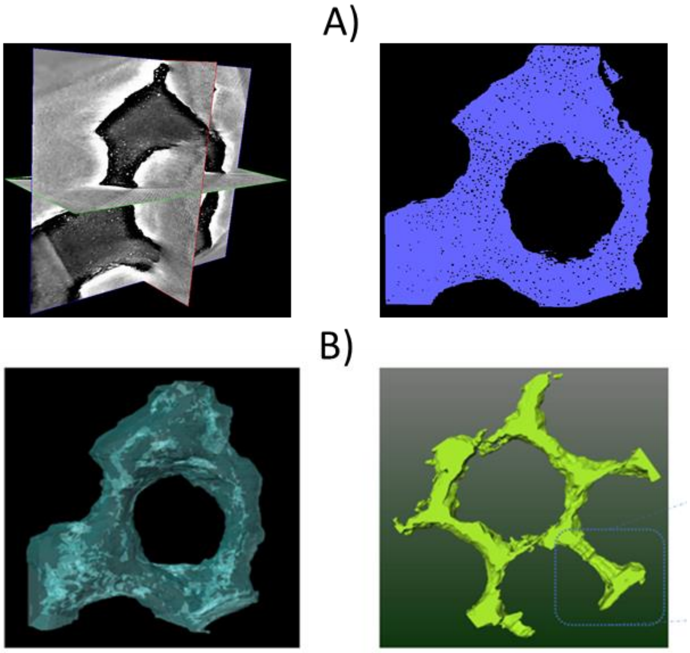

Figure SI7: Electron tomography analysis of the diatomite structure. A) Typical slices showing the presence of the porosity in the analysed areas. B) 3D models illustrating the general shape of a fragment chosen at the level of areola chambers and a global view of the structure.

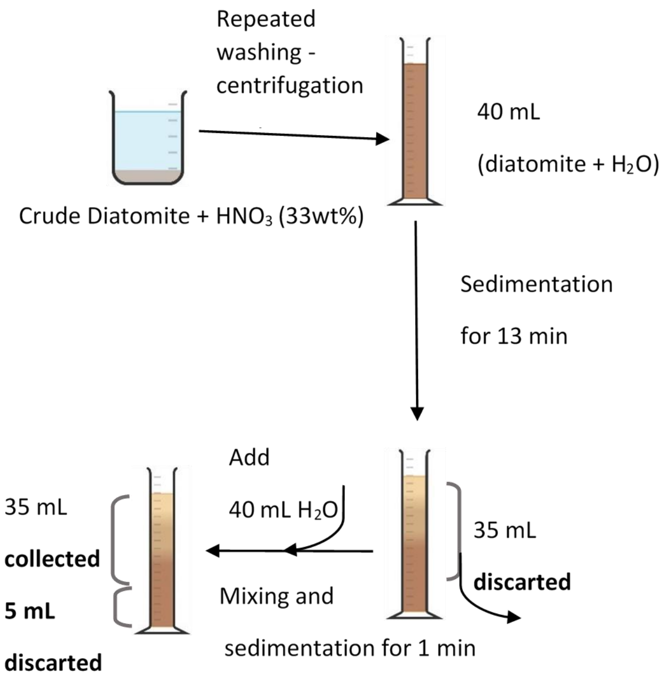

Figure SI8: The protocol for the sample preparation used for obtaining the diatoms frustules.

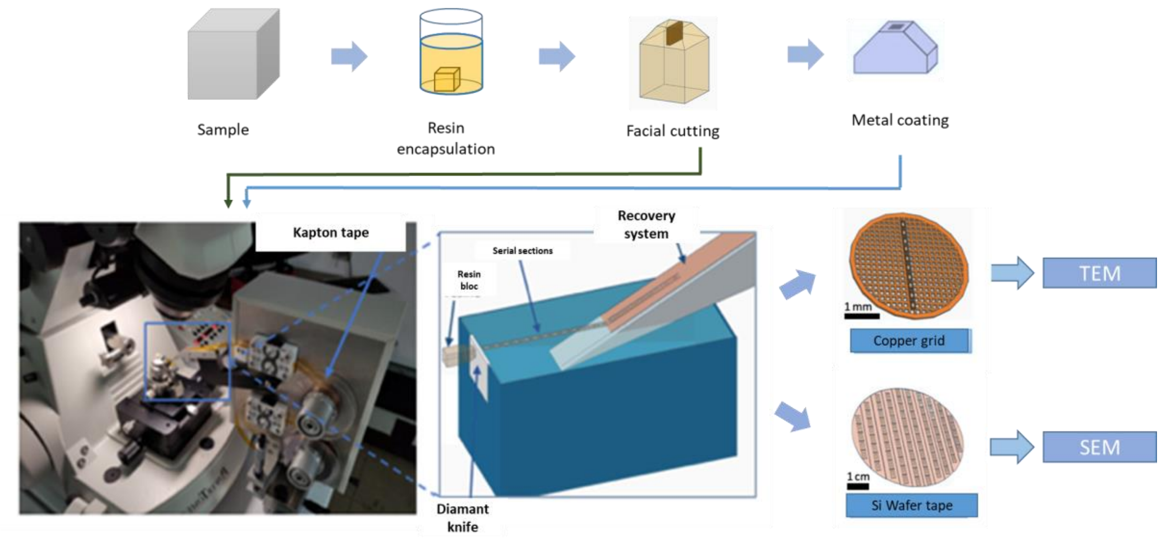

Figure SI9: Protocol used for the preparation of the serial sections by ultramicrotomy, using the ATUM-Tome set-up.
